# Understanding Europe’s skills shortage: Patterns of participation in formal and non-formal education and training

**DOI:** 10.1007/s11159-025-10175-0

**Published:** 2026-03-22

**Authors:** Sarah Pemberton-Frings, Sonia Ilie

**Affiliations:** Faculty of Education, University of Cambridge, Cambridge, UK

**Keywords:** Adult education, Job-related learning, Education and training, Cross-national comparisons, Reskilling and upskilling, Lifelong learning

## Abstract

Technological advancements, environmental commitments and demographic shifts are rapidly reshaping Europe’s labour markets, and thus the skills needed to succeed within them. These changes are driving growth in some industries while leading to contractions in others. Adult learning, both in formal and non-formal contexts, provides pathways for reskilling and upskilling, which are critical for adapting to these shifts. Recognising the importance of education and training, the European Union (EU) set a target that, by 2025, at least 47 per cent of adults aged 24–64 should have participated in some form of learning, education or training during the last 12 months. Using data from the EU Labour Force Survey (EU-LFS), which ran from 2013 to 2022, this article explores progress towards that target, specifically examining the relationship between personal and work-related factors and participation in different forms of learning. The results show that participation in adult education and training has grown significantly, particularly in job-related, non-formal training. Across countries and over time, women participate at higher rates than men in all forms of learning. After adjusting for potential confounding factors, including gender, training rates were found to be highest in highly skilled occupations and lowest in low-skilled occupations. Additionally, individuals in industries with significant skills mismatches are more likely to participate in learning. The analysis indicates progress in addressing skills shortages but raises doubts about whether EU countries have met the 2025 target. These findings underscore the need for targeted policies to address participation in learning, education and training and to support the upskilling and reskilling necessary for the evolving European labour market.

**Supplementary Information:**

The online version contains supplementary material available at 10.1007/s11159-025-10175-0.

## Résumé

Comprendre la pénurie de compétences en Europe : schémas de participation à l’éducation et à la formation formelles et non formelles – Les avancées technologiques, les engagements environnementaux et les mutations démographiques transforment rapidement les marchés du travail en Europe et, par conséquent, les compétences nécessaires pour y réussir. Ces changements stimulent la croissance de certaines industries tout en entraînant des contractions dans d’autres. L’apprentissage des adultes, tant formel que non formel, offre des voies pour la reconversion et la montée en compétences, essentielles pour s’adapter à ces évolutions. Reconnaissant l’importance de l’éducation et de la formation, l’Union européenne (UE) a fixé un objectif : la participation d’ici 2025 d’au moins 47 % des adultes âgés de 24 à 64 ans à une forme d’apprentissage, d’éducation ou de formation au cours des 12 derniers mois. En s’appuyant sur les données de l’Enquête sur les forces de travail de l’UE (EFT-UE), couvrant la période de 2013 à 2022, le présent article examine les progrès réalisés pour atteindre cet objectif, en analysant spécifiquement les liens entre les facteurs personnels et professionnels, et la participation à différentes formes d’apprentissage. Les résultats montrent que la participation des adultes à l’éducation et à la formation a augmenté considérablement, en particulier dans le domaine des formations non formelles liées à l’emploi. Dans tous les pays et au fil du temps, les femmes prennent part davantage que les hommes à toutes les formes d’apprentissage. Après ajustement des facteurs de confusion potentiels – dont le genre –, les taux de formation sont les plus élevés au sein des professions hautement qualifiées, tandis que les plus bas se situent parmi les activités à faible niveau de qualification. Par ailleurs, les individus travaillant dans des secteurs fortement touchés par l’inadéquation des compétences sont plus susceptibles de participer à des activités d’apprentissage. L’analyse indique des progrès dans la lutte contre les pénuries de compétences, mais interroge sur la capacité des pays de l’UE à atteindre l’objectif de 2025. Ces résultats soulignent la nécessité de disposer de politiques ciblées promouvant la participation à l’apprentissage, à l’éducation et à la formation, afin d’encourager la montée en compétences et la reconversion, nécessaires sur marché du travail européen en pleine évolution.

## Introduction

The European labour market is experiencing significant skills gaps, driven by technological, environmental and demographic changes (EC 2024). 2023 was designated the “European Year of Skills”, yet in the same year, more than three quarters of companies in the European Union (EU) reported difficulties in finding workers with appropriate skills (Yanatma 2024). Additionally, 42 per cent of small and medium-sized enterprises indicated a shortage of qualified staff (EC 2023a). The European Skills and Jobs Survey (ESJS) found that over two thirds of workers in several industries reported needing, to a “great” or “moderate” extent, to develop their overall level of knowledge or skills to perform their main job (Cedefop 2021).

To address these skills shortages, the EU set targets related to adult learning. Most prominently, the EU set a target that, by 2025, at least 47 per cent of adults aged 24–64 should have participated in learning, education or training during the last 12 months (CoEU 2021), building on the previous target of 15 per cent in 2020.

To support training participation and provision, the EU implements programmes such as the 2021–2027 EUR 142.7 billion European Social Fund Plus (ESF+)^1^ and aims to enhance the visibility and value of skills. For example, it has proposed that corporate sustainability reporting requirements for large companies include information on training and skills development (EC 2023b).

It is crucial to examine not only the overall rate of participation in learning, but also the heterogeneity of participation. For example, workers in low-skilled occupations face higher automation risks (Cedefop 2019a). These workers require reskilling or upskilling to adapt and transition to new roles. Additionally, industries are evolving at different paces and scales as they digitise, become less carbon-intensive, and implement stricter regulatory compliance, leading to varying demands for new skills across industries. Education and training are essential for workers seeking to meet the evolving needs of their industries.

The study we present in this article examines the heterogeneity of adult education and training by exploring cross-national participation patterns in different adult learning activities. It reviews personal and work-related characteristics with a particular focus on occupation and industry, given their relevance to the skills gap and Europe’s upskilling and reskilling needs.

Our study provides a timely and comprehensive review of participation in various forms of learning, education and training, offering a novel perspective by focusing on occupation and industry. Given the designation of 2023 as the “European Year of Skills” amid a persistent problem of skills mismatch, this research provides up-to-date analysis for timely policy action. It also builds on a rich tradition of cross-national adult learning reviews using the EU Labour Force Survey (EU-LFS).^2^ For example, Norman Davis (1996) used the 1994 LFS to show that training opportunities decline with age and are lower for low-skilled or unskilled workers and the unemployed who are already disadvantaged in the labour market. Maarten Wolbers (2005) used the 1993–2001 LFS to show that countries with strong vocational education systems had higher rates of participation in continuing education and training than countries that focused mainly on general education. Miroslav Beblavy et al. (2014) used the 2000, 2005 and 2010 EU-LFS surveys to show that participation in education and training increases across the life cycle for every age group. Eve-Liis Roosmaa et al. (2019) and Roosmaa and Ellu Saar (2010) used the 2014 EU-LFS to review vocational upper secondary education and participation in non-formal education, and the 2003 EU-LFS to review the relationship between institutional factors and non-formal learning. Using the EU-LFS data collected 2013–2022; our own study contributes a novel perspective by analysing current trends and determinants of adult education and training participation through the lenses of occupation and industry, emphasising the urgent need to address the skills gap in today’s labour market.

Although our analysis draws on data from the EU-LFS, its findings may be relevant to countries beyond the EU. Countries in other world regions are also affected by trends such as rapid technological change, ageing populations, migration and decarbonisation (OECD 2021; WEF 2023), which are reshaping labour markets globally and increasing the need for adult learning systems that support reskilling and upskilling. Therefore, patterns we uncover in this analysis, such as unequal participation in training across occupations, industries and demographic groups, are likely to be of concern in many contexts beyond the EU. As such, our research contributes to a growing international evidence base necessary for designing learning systems that promote equitable access to reskilling and respond to structural labour market transitions.

In this article, we divide learning, education and training into formal and non-formal categories, with the latter further subdivided into job-related and non-job-related learning. Like the EU-LFS, we use the classification of learning activities to define formal and non-formal forms of learning as follows: (1) *formal learning* is institutionalised and recognised by relevant national education or equivalent authorities; (2) *non-formal learning* refers to institutionalised activities not recognised by national education or equivalent authorities (Eurostat 2016). However, the distinction between non-formal and formal education is sometimes contested (Rubenson 2019), with some scholars describing it as artificial and inconsistently applied. To address this, this article presents both the results for overall participation in education and training and trends by specific learning types.

Two additional concepts require definition: upskilling and reskilling. *Upskilling* involves acquiring additional skills in a specific domain to enhance employability and adaptability (Li 2022), helping individuals meet the changing demands of the labour market by building upon their existing knowledge and competencies. For example, upskilling may involve acquiring digital literacy or data analysis skills that are in high demand (Cedefop 2010).

By contrast, *reskilling* involves a more substantial transformation in an individual’s skillset and career trajectory. It requires acquiring new skills to transition into a different occupation or industry (Li 2022). Reskilling becomes necessary when existing skills become obsolete due to technological advancements, industry shifts or changing labour market demands (OECD 2019). This process may involve developing entirely new skills or building upon existing ones to meet the requirements of alternative roles (Li 2022).

In this article, we use “learning” and “education and training” interchangeably to refer to adult participation in both formal and non-formal activities that may facilitate reskilling or upskilling in the context of employment.

The next section provides an overview of the literature related to adult learning, followed by a description of the data and methods we used. The results section examines trends and patterns of learning participation. Finally, the conclusions synthesise the findings from the different forms and determinants of adult learning, education and training, and consider their implications for policy.

## Literature review

### Personal and work-related determinants

A range of personal and work-related characteristics has been associated with participation in adult learning. The most prominent and established of these is the relationship between prior educational attainment and participation (Dieckhoff and Steiber 2011; Wolbers 2005). The “Matthew effect”^3^ suggests that individuals with higher levels of educational attainment are more likely to continue engaging in further education (Boeren 2009). For formal adult education, low prior attainment can pose a barrier to entry. Psychological factors, such as negative experiences of schooling, can also indirectly hinder participation (Field 2006; Illeris 2003; Rubenson and Desjardins 2009). For non-formal adult learning, employer incentives are crucial, as employers often sponsor learning after individuals have entered the labour market. Some scholars suggest that individuals with higher education are also more trainable, so each unit of training yields greater productivity gains for highly educated workers compared to those with lower educational attainment (Boeren 2009; Dieckhoff 2007).

Beyond educational attainment, other personal and work-related factors associated with participation include gender (Acker 1990; Daemmrich et al. 2016), age (Fouarge and Schils 2009; Heckman 2000), presence of children in the home (Dieckhoff 2007; Zoch 2023), marital or co-habitating partner status (Bianchi et al. 2000; O’Connell and Byrne 2012), urbanicity (Bennett and Errington 1990; Henderson 2005), job tenure (Lindsay et al. 2013), hours worked per week or full-time versus part-time status (Albert et al. 2010), contract type (permanent versus temporary) (Ortiz 2010), remote work arrangements (Radovan 2024), and enterprise size (Brunello 2001; O’Connell and Byrne 2012).

This article focuses on occupation and industry. Cecilia Albert et al. (2010) found that individuals working in high-skilled, non-manual occupations across Europe are more likely to participate in training. Wolbers (2005) found that professionals and technicians participated at higher rates than semi- or unskilled manual workers, confirming findings from a decade earlier (Davis 1996) and demonstrating that opportunities for participation were greater in European countries with stronger vocational educational traditions. Similarly, Steven Groenez (2007) found that occupations requiring more intensive and continuous upskilling exhibited higher participation rates.

Occupation is closely related to other factors influencing participation in adult learning. For example, occupation and educational attainment are linked; occupations typically held by highly educated individuals are more often knowledge-intensive, requiring continuous updating of knowledge and skills (Kilpi-Jakonen et al. 2015), whereas low-skilled jobs may remain relatively stable in terms of their required tasks (OECD 2019). Additionally, women in Europe face disadvantages when employed in male-dominated occupations (Dieckhoff and Steiber 2011; Wotschack 2019). There are also cumulative effects of multiple disadvantages for participation in adult learning and education – for example, women with low levels of education tend to work in low-skilled jobs (Kim 2020).

Employment sector is also a key factor shaping training opportunities. Workers in certain industries, such as finance and insurance, are more likely to participate in training regardless of other characteristics (Albert et al. 2010). While participation rates are generally higher in the public sector across most European countries, private-sector industries with well-established, highly formalised training and professional development structures, such as construction, the energy sector and financial services, have substantially higher participation rates (Lindsay et al. 2013). The mechanism by which occupation and industry influence motivation for adult participation in learning was recently examined in a regional German study (Morris et al. 2022), which found that occupational integration, attendance, in-service training, and support in terms of time and funding were all associated with participation in adult learning.

At the onset of COVID-19 lockdowns, Ellen Boeren et al. (2020) predicted that trends by occupation and industry might become affected by the pandemic. Many frontline businesses – particularly small and medium enterprises – in the restaurant, retail, transportation, supply chain, sales and leisure sectors had to lay off employees, requiring them to return to education, upskill, reskill or seek new occupations. Using data from 2013 to 2022, our analysis captures trends in the immediate aftermath of the pandemic, contextualised by pre-pandemic participation patterns.

### Institutional and macroeconomic factors

Several studies have examined institutional effects on participation in training (Blossfeld 2003). Wolbers (2005) found that labour market institutions affected participation, while Roosmaa and Saar (2010) reviewed how the structure of educational systems affects the training gap between low-skilled and highly skilled workers and how different institutional systems have shaped opportunities for lifelong learning in Europe. They found that countries with the highest levels of participation and lowest levels of inequality are coordinated market economies with a well-developed welfare state, while the lowest participation rates were found in “Mediterranean market economies” with weak welfare states (Southern Europe) and in “dependent market economies” with liberal welfare states (e.g., Baltic countries). Hans-Peter Blossfeld et al. (2014) found that higher public expenditure in education was associated with increased participation in formal and employer-sponsored non-formal education.

Ellu Saar and Mari Räis reviewed participation in job-related training in Europe and noted that the United Kingdom (UK), as a liberal market economy, relies mainly on the private sector to provide skills training (Saar and Räis 2017). This is mainly due to the limited vocational focus of the UK’s education system (Green et al. 2015; Wolbers 2005). The labour market does not exhibit the same insider–outsider dynamic seen in countries such as Germany, so employment risks are spread more evenly across the workforce, and individual attributes play a significant role in determining individuals’ labour market success (DiPrete et al. 1997). The relatively low level of employment protection in the UK also places the onus on individuals to enhance their human capital (Estevez‐Abe et al. 2001).

Participation rates also vary depending on macroeconomic conditions. Blossfeld et al. (2014) analysed 26 European countries and found that higher overall unemployment rates were associated with lower probabilities of attending employer-sponsored adult learning activities. This contrasts with the research of Andrea Bassanini et al. (2005), who found higher unemployment rates to be associated with higher adult learning participation.

This article therefore explores the above and further patterns of formal and non-formal learning, education and training participation for a large set of European countries over time. Next, we outline our methodological approach.

## Methods

### Research questions (RQs)

We conducted our study in 2024. Given the context of this research and the availability of data from 2013 and 2022, we formulated our first research question as follows:

RQ1 *To what extent is the EU on track to meet its target that by 2025 at least 47% of adults aged 24–64 should have participated in learning, education or training during the last 12 months?*

Next, considering the heterogeneity of training engagement and the varying rates at which occupations and industries are evolving – and thus experiencing different levels of skills mismatches – we formulated RQ2 and RQ3 as follows:

RQ2 *How does occupational skill level relate to participation in learning, education or training?*

RQ3 *How are individuals in industries with different skills mismatches participating in learning, education or training?*

To address RQ2, we categorised occupations as highly, medium-, or low-skilled according to the skill level classification of the International Labour Organization (ILO 2008).

For RQ3, we had various options for identifying industries with the highest skills mismatches. For example, Eurofound’s European Company Survey (ECS)^4^ asks employers whether they “encounter difficulties in finding employees with the right skills”; however, ECS data are not collected frequently. Similarly, the European Business and Consumer Survey (EU-BCS) asks whether labour shortages are a major factor limiting production, but it only surveys employers in manufacturing, services and construction.^5^ For our own study, we used the European Skills and Jobs Survey (ESJS) conducted in 2021,^6^ which provides a close temporal match to the EU-LFS data. Additionally, it includes comparable industry classifications, making it the most appropriate data source for our analysis. Further details are provided in the *Measures* section below.

### Data

As mentioned in the introduction, the study we present here used data from the European Union Labour Force Survey (EU-LFS). This comprehensive household survey provides insights on labour participation for individuals aged 15 and above, including those outside the labour force, across the 27 EU Member States and three of the four European Free Trade Association (EFTA) Member States (Switzerland, Iceland and Norway). The annual sample size exceeds one million respondents and covers all industries and occupations, enabling analysis of how access to learning correlates with personal and work-related attributes. The 2022 EU-LFS dataset – the most recent available at the time of writing – served as the primary basis for this research, with earlier survey waves from 2013 also included.

National statistical institutes across Europe are responsible for sample selection, questionnaire preparation, household interviews, and the compilation and submission of data to Eurostat. Data comparability across countries and over time is assured by using consistent concepts and definitions, adhering to ILO guidelines, applying common classifications (e.g., NACE),^7^ and collecting data based on the same set of characteristics in each country. The datasets include weighting variables to ensure representativeness and to adjust for potential sampling biases.

### Sample

Our analysis focuses on individuals aged 25–64, aligning with the EU target specification relevant to RQ1 and concentrating on those most likely to be engaged in the workforce, rather than individuals who may still be in tertiary education and not yet employed. Table 1 details the sample size we used in our analysis. While the age range 25–64 was chosen to reflect typical workforce participation, it is a limitation, as in certain regions – especially Southern Europe – the demographic engaged in tertiary education may be older, with many individuals concurrently employed during their studies (OECD 2023).

**Table 1:** Number of male and female respondents aged 25–64 in selected European countries (EU-LFS 2022)

	Male	Female		Male	Female		Male	Female
AT	47,201	49,153	ES	21,272	22,893	LV	2,147	2,593
BE	9,725	10,354	FI	8,815	8,993	MT	2,838	2,780
BG	8,128	7,976	FR	15,739	17,463	NL	82,401	84,363
CH	22,063	23,201	HR	10,042	10,480	NO	11,217	11,152
CY	9,443	11,045	HU	54,072	56,001	Pl	72,216	77,737
CZ	8,976	9,449	IE	4,956	5,474	PT	7,795	8,755
DE	52,705	52,963	IS	3,762	4,153	RO	56,036	57,367
DK	19,448	21,881	IT	119,862	130,418	SE	35,176	34,475
EE	6,171	6,870	LT	12,711	16,182	SI	17,759	18,344
EL	6,947	7,367	LU	5,359	5,591	SK	20,211	21,668

### Measures

Our study examined participation across five types of adult learning: (1) overall participation in education or training during the past 12 months, and disaggregated participation in (2) formal education, (3) non-formal education, (4) non-formal job-related learning, and (5) non-formal non-job-related learning (see Table 2).

**Table 2:** Types of learning, education and training

Type of education & training	Description
All^*a*^	Participation in education or training in the previous 12 months/4weeks
Formal	Participation in formal education (student or apprentice) and training in the last 12 months/4 weeks
Non-formal^*b*^	Participation in non-formal education and training in the last 12 months/4 weeks
Non-formal, job-related	Participation in at least one job-related non-formal education or training activity in the last 12 months/4 weeks
Non-formal, non-job-related	Participation only in non-job-related/personal non-formal education or training activities in the last 12 months/4 weeks

*Notes*: ^*a*^Includes formal and non-formal learning; ^*b*^includes job-related and non-job-related learning

The core explanatory variables are occupation and industry. As previously noted, occupation is categorised according to the ILO skill level classification (ILO 2008), which distinguishes four groups: (0) armed forces occupations; (1) low skill level (elementary occupations); (2) mid skill level (clerical support workers; service and sales workers; skilled agricultural, forestry and fishery workers; craft and related trades workers; plant and machine operators, and assemblers); and (3) high skill level (managers; professionals; technicians and associate professionals).

We defined industry-level skills mismatch using data from ESJS; industries in which 66 per cent or more of respondents reported a strong or moderate need to upskill are classified as high-mismatch industries (Cedefop 2021). These include professional services, information and communication, finance and insurance, mining and quarrying, education, energy supply services, and public administration and defence.

Other personal and work-related characteristics were included due to their established association to adult learning participation in the literature. Personal characteristics include sex (male, female), age group (16–24, 25–34, 35–44, 45–54, 55–64), highest level of educational attainment (lower secondary, upper secondary, third level), presence of children in the household (yes/no), co-habitation status (spouse or partner in same household vs not), and urbanicity (cities, towns and suburbs, rural areas). Age was measured as a categorical variable due to data availability limitations in some countries.

Work-related characteristics include job tenure (<1 year; 1–5 years; 5–10 years; ≥10 years), employment status (full-time vs part-time), contract type (permanent vs fixed-term), frequency of working from home (mainly, sometimes, never), employer size (<10; 10–19; 20–49; 50–249; 250+ employees), weekly working hours (<30; 30–50; 50–80; 80+ hours), and number of jobs held (one; two; three or more). While the number of jobs is not widely used in prior studies, we included it in our own analysis because holding multiple jobs may constrain participation due to scarcity of time or reflect employment precarity, potentially influencing access to or uptake of training opportunities.

All variables were measured at the individual level. Full coding specifications are provided in the Supplementary Online Appendix (SOA Table 1).^8^

### Statistical modelling and approach

We began our analysis with a descriptive exploration of participation rates in overall, formal, non-formal, non-formal job-related, and non-formal non-job-related education and training, focusing on trends over time and across industries and occupations.

We then used a survey-weighted logistic regression model to examine personal and work-related determinants and the likelihood of participating in education or training, structured as:

$$logit\left({P}_{i}\right)=\mathrm{ln}\left(\frac{{P}_{i}}{1-{P}_{i}}\right)={\beta }_{0}+{\beta }_{1}{age}_{i}+{\beta }_{2}{sex}_{i}+{\beta }_{3}{educational attainment}_{i}+\dots +{\beta }_{n}{X}_{n,i}$$

where

${P}_{i}$ is the probability that individual *i* participates in (1) any education and training, (2) formal education, (3) non-formal education, (4) job-related non-formal education, or (5) non-job-related non-formal education.

${\beta }_{0}$ is the intercept.

${age}_{i}$, ${sex}_{i}$, ${educational attainment}_{i}$, … ${X}_{n,i}$ are the personal and job-related predictors for individual *i*.

${\beta }_{1}$ to ${\beta }_{n}$ are the coefficients for each independent variable (age, sex, etc).

Consistent with the sample description above, we ran the model for the population aged 25–64 in each country to account for country-specific trends and heterogeneity.

### Missing data

When variables were unavailable, we conducted the regression for that country and year excluding that variable. A list of missing variables is summarised in Table 3.

**Table 3:** Variable issues by country and year

Country	Year	Variable/issue
IS	2022	Issue in coding of non-formal education & training affecting 2022, likely overestimated
IS	2022	Child and partner variables missing, regression run without these variables
CH	2013–2022	
NL	2013–2021	
NO	2013–2021	
NL	2022	60% of observations lack data for the child and partner variables for the 25–64 age group, so the regression analysis was run without these variables
SI	2013–2022	Employer size variable missing Number of hours worked only includes hours from main job (no values for hours worked in second job)
DE	2018	Employer size coded incorrectly in the original data (known issue)

## Results

This section presents the empirical findings addressing our study’s three research questions. The results are structured into three parts. First, we present our analysis of temporal trends in adult learning participation from 2013 to 2022 to assess progress towards the EU target (RQ1). Second, we examine cross-sectional descriptive patterns from 2022 by country, gender, occupation and industry to contextualise participation inequalities (RQ2 and RQ3). Third, we use survey-weighted logistic regression models to isolate the effects of occupation and industry on participation in different types of learning – while accounting for other personal and job-related factors.

### Temporal context: participation in education and training from 2013 to 2022

RQ1 asks about progress towards the EU’s 2025 target of 47 per cent adult participation in education and training. As shown in SOA Figure 1 in the Supplementary Online Appendix, participation rates generally increased between 2013 and 2022 (from an average of 12% to 15%), with the most notable growth occurring in 2020–2022. The surge in learning during the COVID-19 pandemic likely reflects multiple overlapping dynamics. Job loss and economic disruption may have prompted some individuals to seek new skills to transition into more resilient or in-demand occupations. Similarly, the shift to remote work may have freed up time and reduced logistical barriers to participation, particularly for white-collar workers. Economic uncertainty may also have acted as a catalyst for some to invest in training to enhance job security and labour market competitiveness, while simultaneously deterring others – particularly those in precarious employment – from engaging in learning due to financial or psychological strain.

**Figure 1:**
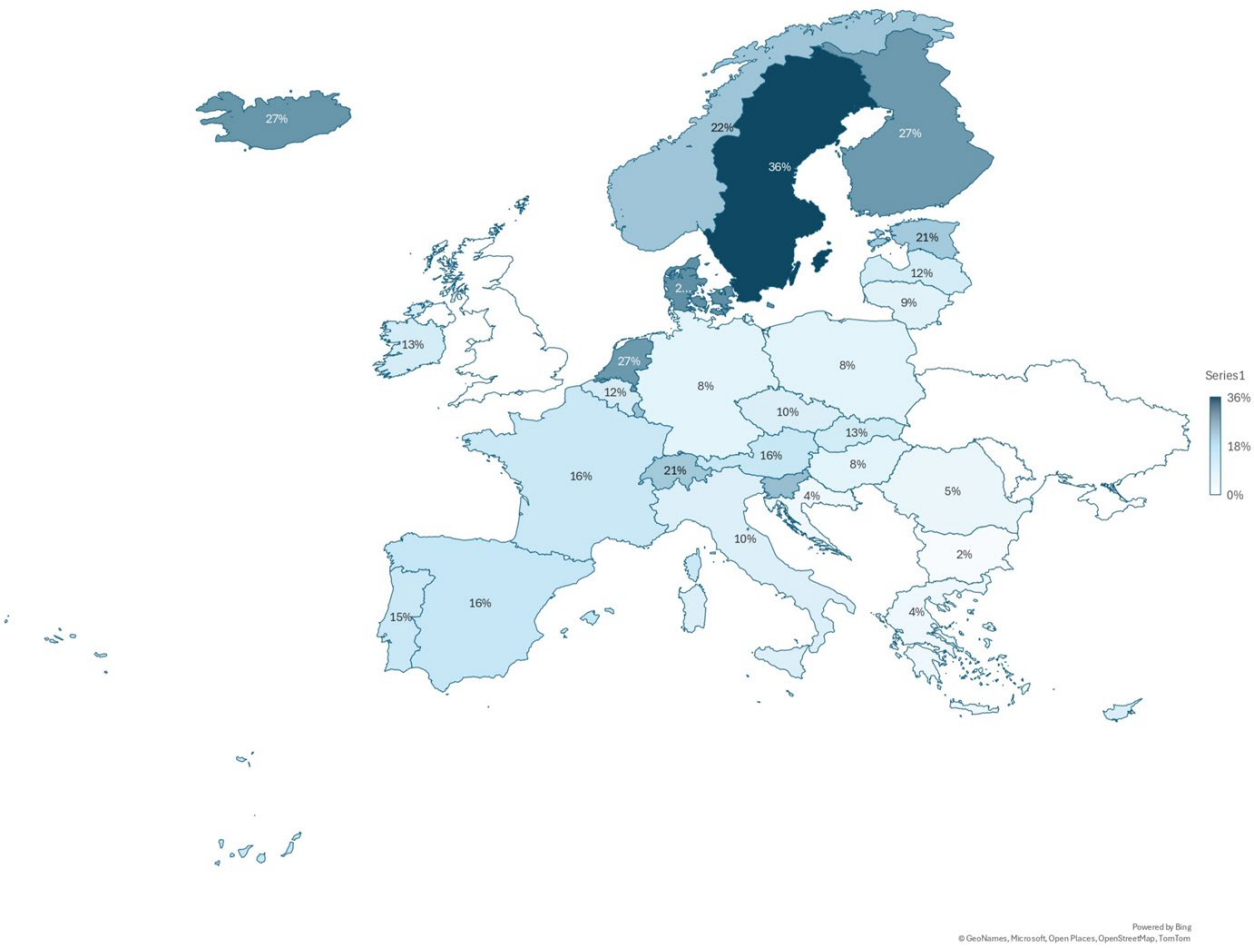
Participation in all education and training in selected European countries in 2022

However, the sustainability of the growth in participation during 2020–2022 remains uncertain. Table 4 shows participation rates in 2022 and estimates the years needed to reach the 47 per cent target based on both the 2013–2022 and 2020–2022 trends. Notably, only Sweden appeared likely to meet the EU’s goal of 47 per cent participation by 2025. Additionally, the negative trend over 2013–2022 for some countries with relatively high baseline participation (e.g., Denmark and Switzerland) suggests possible stagnation, indicating that further increases in participation may prove difficult to achieve under policy and systemic conditions in place at the time of data collection. Overall, despite the upward trends in most countries, EU-wide progress remains uneven, thus the achievement of the 47 per cent target seemed unlikely.

**Table 4:** Latest participation rates in all education and training in selected European countries, and estimated years needed to reach the 47 per cent target based on recent trends.

Countries		Participation rate in 2022, weighted %	Years needed to reach 47% participation	
			2013–2022 rate	2020–2022 rate
AT	Austria	16	168	15
BE	Belgium	12	62	12
BG	Bulgaria	2	6796	296
CH	Switzerland	21	Negative trend	Negative trend
CY	Cyprus	10	101	13
CZ	Czech Republic	10	Negative trend	15
DE	Germany	8	Negative trend	89
DK	Denmark	28	Negative trend	5
EE	Estonia	21	28	12
EL	Greece	4	302	234
ES	Spain	16	78	13
FI	Finland	27	99	Negative trend
FR	France	16	Negative trend	38
HR	Croatia	4	292	69
HU	Hungary	8	74	27
IE	Ireland	13	61	42
IS	Iceland	27	223	6
IT	Italy	10	97	30
LT	Lithuania	9	129	56
LU	Luxembourg	22	30	9
LV	Latvia	12	55	11
MT	Malta	14	44	19
NL	Netherlands	27	21	6
NO	Norway	22	1079	10
PL	Poland	8	108	20
PT	Portugal	15	55	12
RO	Romania	5	108	19
SE	Sweden	36	13	3
SI	Slovenia	22	23	4
SK	Slovakia	13	32	7

This modest overall increase was driven almost entirely by growth in non-formal learning. On average, participation in formal education remained stagnant at around four per cent, with few country-level fluctuations exceeding one percentage point between 2013 and 2022. By contrast, non-formal learning rates rose from an average of nine per cent in 2013 to 12 per cent in 2022. The persistence of low participation in formal learning may indicate institutional inflexibility or a lack of integration between higher education and labour market needs. Conversely, the growth of non-formal training, particularly in 2020–2022, suggests that these modes may be more responsive to immediate labour market pressures. Opportunities to better align formal learning with labour market demands and to sustain or accelerate the growth of non-formal learning are discussed under *Recommendations* at the end of this article.

### Cross-sectional descriptive patterns in 2022

To address RQ2 and RQ3, we first examined descriptive patterns in learning participation by country and gender, before conducting an analysis of occupational and industry participation rates in 2022. This analysis provides context for interpreting the subsequent regression results.

#### Country-level differences

As shown in Figure 1, participation rates in education and training varied widely in 2022, ranging from just three per cent in Bulgaria to 59 per cent in Sweden, with an average of 31 per cent. Nordic countries generally exhibited higher participation rates in all learning categories compared to several Balkan countries (such as Bulgaria, Greece and Romania). This variation aligns with previous research (e.g., Davis 1996; Kilpi-Jakonen et al. 2015; Roosmaa and Saar 2010; Wolbers 2005), which demonstrates the influence of institutional factors on participation in adult learning. Nordic countries, for example, tend to offer generous public support, have strong employer involvement, and place a cultural emphasis on lifelong learning, all of which may contribute to higher participation rates. Conversely, lower rates in Southern and Eastern Europe may reflect weaker adult learning infrastructure or lower public investment in lifelong education. These regional and national differences may also reflect variations in workforce composition, with different countries differing in the representation of occupations or industries in which learning is more prevalent.

For additional detail, see SOA Figure 2 in the Supplementary Online Appendix, which shows participation rates in different types of learning in selected European countries in 2022.

#### Gender differences

In nearly all countries and across all types of learning, women participated at higher rates than men (see SOA Figure 3). This gender gap was most pronounced in non-formal education, where women’s participation exceeded men’s by an average of 2.6 percentage points, compared to 1.6 points for formal learning. The largest gaps were observed in Nordic and Baltic countries (such as Finland, Estonia, Iceland and Sweden), where the gender participation gap exceeded 10 percentage points. These countries are known for stronger gender equality policies and well-developed adult learning systems, which may explain higher female participation. Overall, higher levels of female participation in learning may reflect women’s greater representation in service-oriented industries (e.g., education, health, and social care), where training is more institutionalised and, in some cases, mandated. It may also be linked to higher female enrolment in tertiary education (due to the Matthew effect, as mentioned earlier), or a stronger orientation towards upskilling for career advancement or job security.

Conversely, in countries like Slovakia, the Czech Republic and Italy, men participated at a slightly higher rate than women. In these countries, occupational segregation or care responsibilities may limit women’s access to training. These patterns underscore the importance of analysing gender participation while controlling for occupation and industry (RQ2 and RQ3), which we first explored descriptively and then analysed through regression models. For additional commentary on gender differences by type of learning, see SOA Table 2 in the Supplementary Online Appendix.

#### Participation rates by occupation

SOA Table 3 presents participation rates in formal and non-job-related training by occupation. Addressing RQ2, we found that highly skilled occupations generally have higher participation rates in both formal and non-formal job-related training across most countries. These occupations are more knowledge-intensive and therefore require continuous updating of knowledge and skills (Kilpi-Jakonen et al. 2015), leading to higher training participation rates. By contrast, low-skilled occupations typically have the lowest participation rates in both formal and non-formal job-related training. While this may be anticipated given the relative stability of tasks in low-skilled jobs (Kilpi-Jakonen et al. 2015), it is concerning, as jobs in these occupations are most at risk of automation (OECD 2021).

National contexts moderate the relationship between occupation and training participation. Some countries (e.g., Bulgaria, Greece, Croatia, Poland and Romania) showed comparatively low training rates across occupations, suggesting trans-occupational barriers to learning opportunities. Conversely, Iceland, Norway, Sweden and Slovakia showed relatively high training rates across occupations, indicating the presence of cultural, policy and other country-specific factors that promote learning across occupations. As mentioned above, national average participation rates may stem not only from workforce composition (e.g., concentration in certain industries or occupations) but also from broader educational and institutional environments. These findings emphasise the need to account for differences across countries, occupations and industries (as discussed below) when modelling participation in adult learning, as both national context and workforce composition may influence training participation.

#### Participation rates by industry

In addition to occupation, industry type plays a key role in shaping training participation (SOA Table 4). Industries such as education, human health and social work, and public administration and defence consistently reported higher participation rates across most European countries, potentially reflecting ongoing professional development required in these fields (Cedefop 2019b). Similarly, industries such as information and communication, finance and insurance, and professional, scientific and technical services also demonstrated robust participation across learning types in many countries, potentially driven by technological advancements and regulatory changes characteristic of these industries.

Addressing RQ3, we found that high-participation industries largely overlap with those identified as having the greatest reported upskilling needs. This alignment suggests that higher participation may be a response to rapidly evolving skills demands, with training serving as a strategy to maintain relevance and productivity in fast-changing industries. By contrast, industries such as agriculture, forestry and fishing, and transportation and storage tended to exhibit lower participation rates in both formal and non-formal job-related training. These lower rates may reflect a combination of slower-changing skills demands, limited access to training infrastructure, or a lack of perceived relevance of training within these sectors.

Geographic patterns further contextualise these trends. Northern and Western European countries (e.g., Denmark, Sweden, Norway) generally reported higher participation rates across all forms of education across industries. In contrast, Southern and Eastern European countries (e.g., Bulgaria, Greece, Romania) had lower engagement across industries.

The descriptive findings above reveal patterns by country, gender, occupation and industry, but do not disentangle the effects of overlapping characteristics. To examine RQ2 and RQ3 more closely, and to focus on the roles of occupation and industry as variables of interest, we next present our multivariate regression analysis to strengthen the evidence base for targeted policy design.

### Modelling the combined effects of personal and work-related characteristics on participation in adult learning, education and training in 2022

To isolate the relationship between occupational skill level and learning participation (RQ2), and between industry-level skills mismatch and participation (RQ3), we estimated survey-weighted logistic regressions for participation in different forms of learning.

SOA Table 5 in the Supplementary Online Appendix presents the estimated results of the models on participation in learning among individuals aged 25–64 across the 30 countries in 2022. For brevity, and following the approach of Albert et al. (2010), SOA Table 5 summarises the results rather than presenting all 30 estimated models for each learning type. It indicates, by country and direction (positive or negative), where the coefficient of each independent variable is statistically significant.

#### Occupation and learning participation

Across countries and learning types, occupational skill level emerged as one of the most consistent predictors of participation in learning. Addressing RQ2, we found that workers in highly skilled occupations were significantly more likely to participate in both formal and non-formal learning than those in mid-skilled occupations, whereas low-skilled workers were generally less likely to engage in any form of learning compared to their mid-skilled counterparts.

These findings mirror the descriptive patterns discussed above and align with prior research highlighting skill-biased access to training (Kilpi-Jakonen et al. 2015). They suggest that the structure of occupational roles may influence both access to and demand for training. Highly skilled jobs often require continuous learning and adaptation to evolving technologies or professional standards, making training both more necessary and better supported. In contrast, low-skills occupations tend to have fewer formalised learning pathways, lower perceived returns to training, and less employer investment (OECD 2019). The persistence of this disparity, even after controlling for education, gender, age, and other factors, reinforces the notion that occupation itself – perhaps as a proxy for job complexity, autonomy and employer norms – acts as a gatekeeper for lifelong learning. As Europe navigates major transitions ahead, these occupational divides raise concerns about uneven resilience and adaptability across the workforce.

To contextualise the 2022 findings, we conducted regressions for each country and for each year from 2013 to 2021. The magnitude of the skill-level coefficient for high skills increased over the decade in most countries where that coefficient was significant, suggesting that participation became increasingly tied to skill level. This intensification of the occupational gradient in training participation may indicate a need for greater investment in structured learning pathways, particularly for low-skilled workers; this issue is discussed further under *Recommendations* at the end of this article.

#### Industry and learning participation

Turning to RQ3, we found that the degree of reported skills mismatch in an industry was also significantly associated with learning participation, consistent with the descriptive trends outlined above. In countries with significant coefficients, individuals in industries with high skills mismatches were generally more likely to engage in all types of learning, particularly in non-formal and job-related training. Ireland was an exception to this trend.

The consistency of this relationship across countries suggests that industry-level dynamics may also shape participation in learning. Workers in fast-evolving sectors may seek training in response to awareness of changing skills requirements. Many of these industries, such as ICT, finance and professional services, are characterised by rapid innovation, evolving regulatory standards, and high levels of skills obsolescence. However, reported skills mismatches may also reflect constraints in training availability or effectiveness; high levels of perceived need could indicate a gap between current provision and actual labour market demands. This dual interpretation reinforces the importance of industry-specific monitoring and policy action. For learning systems to support economic transformation and inclusive growth, they must be both responsive to industry skills shifts and equitable in distributing opportunities for adaptation. These considerations are discussed under *Recommendations* at the end of this article.

Over time, the predictive power of industry mismatch was more stable than that of occupational skill level, suggesting that industry learning dynamics have remained consistent, whereas occupational stratification has intensified.

#### Other work-related and personal factors

Beyond occupation and industry, two job-related characteristics – employer size and remote work arrangements – frequently emerged as significant predictors of non-formal training participation. Employees in larger organisations were more likely to participate than those in organisations with fewer than 10 employees, likely reflecting greater institutional support, resources, and formalised training structures. Work-from-home arrangements also played a role; individuals who sometimes worked remotely were more likely to participate in non-formal learning than those who mainly worked from home, while those without any remote work opportunities were significantly less likely to participate in non-formal job-related training. These patterns suggest that flexibility in work location may facilitate access to or engagement in learning, or that companies offering such flexibility may also promote learning participation.

Among personal characteristics, gender was a significant predictor of learning participation in at least one third of countries after controlling for other factors. In those countries, women were consistently more likely to participate in learning activities than men, reinforcing the patterns outlined above. This suggests that women’s higher representation in certain occupations or industries and higher levels of attainment in tertiary education do not fully explain their higher levels of participation in learning. However, comparing our regression results between 2022 and previous years, we find that the magnitude of the relationship between gender and participation decreased over the period 2013–2022, indicating that gender has become less of an explanatory factor.

Age was also significantly associated with participation, particularly in formal learning. In countries where age had a significant effect, individuals in the age cohort 25–34 were more likely than other age cohorts to have participated in formal learning. Those aged 55–64 were less likely to participate in non-formal learning, except in Italy. Notably, in Iceland and Italy, older cohorts were more likely to participate in non-formal, job-related training than those aged 25–34, potentially reflecting targeted training programmes for older workers in these contexts.

We observed a clear and robust relationship between educational attainment and participation in learning: individuals with upper secondary or third-level education were significantly more likely to participate than those with only lower secondary education. Notably, educational attainment was a stronger predictor of participation in non-formal education than of participation in formal education. In half of the countries in our sample, third-level education was more strongly associated with participation in non-formal learning than lower secondary education, whereas in only four countries was it a significant predictor of participation in formal learning.

Collectively, these results confirm substantial heterogeneity in learning participation, and both reinforce and add nuance to findings from literature available at the time of our research. On the one hand, the associations observed for gender, age, educational attainment and the other factors listed above mirror existing research on the determinants of adult learning. On the other hand, other factors cited in past studies, including tenure, part-time vs full-time, permanent vs temporary roles, number of jobs held, having a dependent child at home, having a spouse or cohabiting partner, and living in rural/suburban areas, were significant predictors of participation for all types of learning for less than one third of countries. This divergence suggests that while certain predictors remain robust, others may be diminishing in explanatory power or may reflect patterns better explained by factors such as occupation and industry. The discussion below addresses the implications of these findings.

## Conclusions

The findings from our study illuminate significant trends and disparities in adult learning participation across Europe over the past decade. First, with regard to RQ1, the 2013–2022 descriptive analysis indicates that participation in education and training has generally increased, primarily driven by job-related non-formal training. However, most EU countries appear to have failed to meet the ambitious 2025 goal of having 47 per cent participation in adult learning activities (within the last 12 months). More research is needed to identify the barriers to participation and to implement policies to increase it, especially since personal characteristics, such as gender, and work-related characteristics, such as occupational skill level, are clearly associated with participation over time.

Second, our study found that women participate at higher training rates than men in every type of learning: by 1.6 percentage points in formal learning, by one percentage point in non-formal job-related learning, and by 1.3 percentage points in non-formal, non-job-related learning. Gender was rarely significantly associated with participation in the models, even after controlling for other factors. This trend may reflect women’s higher participation in tertiary education (Eurostat 2023). Those who receive more education tend to seek out further learning opportunities (Boeren 2009), so if women participate in tertiary education more, they may also engage more in other forms of learning once they are employed. After controlling for educational attainment, gender was not statistically significant in most countries. Higher rates of job-related training for women may suggest that women are more likely to seek training when a skills gap is perceived, use training as a tool for advancement, or that training programmes are better tailored to women, possibly designed to close gender gaps in the workplace (Rubiano-Matulevich and Beegle 2020).

Third, with regard to RQ2, both descriptively and in the regression models, we found training participation rates to be highest in highly skilled occupations and lowest in low-skilled occupations. This aligns with the understanding that highly skilled jobs often require continuous skills upgrading to keep pace with technological advancements and changing job demands (Groenez 2007). This disparity highlights a critical area for intervention, as upskilling and reskilling low-skilled workers are essential for reducing skills mismatches and improving employability in a rapidly changing labour market (WEF 2017). Policies that focus on accessible and affordable training for low-skilled workers could help bridge this gap.

Fourth, with regard to RQ3, both descriptively and in regression models, we observed differences in participation rates across industries. Individuals in high-skills-mismatch industries participate more in every form of learning, controlling for other factors. It is encouraging that in industries in which individuals report a strong or moderate need to update their knowledge and skills, training participation rates are also highest. However, other means of identifying which industries would benefit from training should be considered, such as employer surveys or administrative data to identify industries vulnerable to automation, as well as shrinking or carbon-intensive industries in which job characteristics are likely to change. More research is needed to determine whether training rates are sufficient to ensure smooth transitions for workers in these industries.

Our study provides valuable insights into the patterns and determinants of adult learning participation in Europe. The findings underscore the importance of tailoring policy measures to enhance participation rates, address gender disparities, support low-skilled workers, and meet the specific needs of industries facing skills mismatches. However, the above findings are general; regional differences exist. Nordic countries (e.g., Denmark, Finland, Iceland, Norway, Sweden) generally exhibit higher participation rates across all forms of learning than Southern European countries (e.g., Greece, Italy, Portugal, Spain) and Eastern European countries (e.g., Bulgaria, Croatia, Hungary, Romania). This variation aligns with previous research and may reflect differences in policy frameworks, cultural attitudes towards learning, and the availability of training opportunities across these regions (Roosmaa and Saar 2010; Saar and Räis 2017). These regional disparities may provide evidence to support the development of locally effective policies, such as those recommended below.

### Recommendations

The evidence above suggests that addressing the gaps in adult learning participation and skills development requires a targeted approach that recognises each country’s educational system and labour market contexts.

Structural trends such as automation, decarbonisation and digitalisation are likely to affect countries and industries in different ways (Li 2022), partly contingent on the prevalence of industry sectors most at risk from such trends and the skills levels of workers within them. Identifying priority groups for reskilling or upskilling – nationally, regionally and by industry – therefore represents a critical step in aligning education policy with workforce transformation (Ure and Skauge 2019). The identification of priority groups can be informed by data from employee surveys that reflect workers’ perceptions of their ability to meet job demands (e.g., the European Skills and Jobs Survey [ESJS], which measures upskilling needs by industry), employer surveys that assess current and projected skills needs (e.g., Eurofound’s European Company Survey [ECS]) and administrative labour market data (such as the EU Labour Force Survey [EU-LFS] we used for this research, or reports from the Brookings Institution or McKinsey Global Institute, which provide projections) that identify high-level, systemic trends in adult learning participation.

Establishing systems to track and project skills mismatches over time would further strengthen the evidence base for intervention (McGuinness et al. 2018). Singapore’s Future Economy Council (FEC), which tracks and projects skills mismatches to ensure readiness for future changes, offers an example of how national-level tracking mechanisms can inform timely and targeted policy interventions.^9^

At the same time, policymakers need to assess current levels of reskilling and upskilling. While this article documents variation in participation rates, additional research is needed to understand the underlying reasons for underrepresentation among certain groups. Key areas of inquiry include the availability of learning opportunities for certain population groups, the extent to which existing programmes are underutilised, and whether certain groups disproportionately benefit. Each of these issues is considered in detail below.

Where learning opportunities are lacking, policymakers may consider a range of incentives to support training provision. For example, countries may expand on the EU’s Corporate Sustainability Reporting Directive (EP and CoEU 2022), which requires large companies to disclose their contributions to employee training. Financial incentives, such as Singapore’s SkillsFuture Credit,^10^ which allocates training funds directly to individuals, or Canada’s tax credits for employer-sponsored training,^11^ can incentivise training provision and participation. Consideration should be given to the most appropriate providers and delivery mechanisms – whether universities, private employers, or public–private partnerships – to ensure programmes align with national and industry-specific needs (Mullins et al. 2016). In cases where scaling is feasible, this may include exploring the potential to scale platforms that facilitate the sharing of learning resources among individuals and companies (Rosendale and Wilkie 2020), supported by government or employer subsidies for high-demand skills related to fields such as AI, green technologies or data analysis.

Where learning opportunities exist but participation remains low, more research is needed to identify barriers (Roosmaa and Saar 2017). This could include exploring the focus and level of training content, industry relevance, provider, and the relationship to employment, both informal and formalised through training programmes. This may also involve standardising or certifying different learning opportunities to clarify what a training programme entails for providers, participants and employers (Kato et al. 2020; Mitchell et al. 2025; Ward et al. 2021). Such measures may reduce the friction associated with demonstrating skills when transitioning between jobs or sectors.

In contexts where participation is unequal across groups, assessing the inclusivity and accessibility of training content and delivery may suggest effective group-specific initiatives. For example, Ireland’s Women ReBOOT programme supports women re-entering the technology sector, addressing gender disparities in male-dominated fields.^12^ Similarly, Finland’s adult learning programmes include language options and hybrid or online delivery models to improve accessibility for workers in remote areas or with mobility constraints.^13^ Finally, advances in technology may enhance access to training and personalisation; for example, Singapore’s One Talent Gateway leverages AI to assess individuals’ current skill levels and recommend personalised learning pathways.^14^

Finally, robust monitoring and evaluation of training initiatives are critical to creating effective programmes that improve employment outcomes. Regular monitoring of interventions by governments and stakeholders – including participation rates, skills acquisition and workforce outcomes – may help ensure responsiveness and relevance of training systems over time. For example, Canada’s Future Skills Centre (FSC)^15^ evaluates the effectiveness of training programmes and provides evidence-based recommendations to policymakers. By adopting a dynamic approach, training initiatives can align with labour market needs and adapt to technological, environmental and demographic changes, fostering a more inclusive and adaptable workforce across Europe and beyond.

## Supplementary Information

Below is the link to the electronic supplementary material.

Supplementary file1 (PDF 432 KB)

## Footnotes

1 According to the European Commission’s dedicated ESF+ website, this fund “is the European Union (EU)’s main instrument for investing in people and supporting the implementation of the European Pillar of Social Rights”. For more information, visit https://european-social-fund-plus.ec.europa.eu/en/what-esf [accessed 25 February 2026].

2 The EU-LFS, a large household sample survey, has been collecting data since 1983, providing quarterly results on labour participation of people aged 15 and over and on people outside the labour force. For more information, visit https://ec.europa.eu/eurostat/web/microdata/european-union-labour-force-survey [accessed 25 February 2026].

3 This refers to a phenomenon where individuals or groups who already possess status, resources or recognition tend to gain more over time, while those with less tend to fall further behind, resulting in cumulative inequality. The term was coined by sociologists Robert K. Merton (1968) and Harriet Zuckerman (1977) and is named after a passage in the biblical Gospel of Matthew (25:29): “For to everyone who has, more will be given … but from the one who has not, even what he has will be taken away.”

4 According to Eurofound’s dedicated website, “The European Company Survey (ECS) has been carried out regularly since its inception in 2004–2005 as the European Establishment Survey on Working Time and Work–Life Balance (ESWT). The second survey, under the new title European Company Survey, was completed in 2009, the third in 2013 and the fourth in 2019 in collaboration with Eurofound’s sister agency Cedefop.” For more information, visit https://www.eurofound.europa.eu/en/surveys-and-data/surveys/european-company-survey [accessed 27 February 2026].

5 For more information about the European Business and Consumer Survey (EU-BCS), visit https://ec.europa.eu/eurostat/web/euro-indicators/information-data/business-consumer-surveys [accessed 27 February 2026].

6 For more information about the European Skills and Jobs Survey (ESJS), visit https://www.cedefop.europa.eu/en/projects/european-skills-and-jobs-survey-esjs [accessed 27 February 2026].

7 NACE refers to the “statistical classification of economic activities” in the European Community. The acronym is the abbreviation of the French designation, “*Nomenclature statistique des activités économiques dans la Communauté européenne*”. For more information, visit https://ec.europa.eu/eurostat/web/nace [accessed 27 February 2026].

8 The Supplementary Online Appendix for this article is available at https://doi.org/10.1007/s11159-025-10175-0.

9 For more information about Singapore’s Future Economy Council (FEC), see CAAS (2025).

10 For more information about Singapore’s SkillsFuture Credit, visit https://www.myskillsfuture.gov.sg/content/portal/en/career-resources/career-resources/education-career-personal-development/SkillsFuture_Credit.html [accessed 27 February 2026].

11 For more information about the Canada training credit (CTC), visit https://www.canada.ca/en/revenue-agency/services/child-family-benefits/canada-training-credit.html [accessed 27 February 2026].

12 For more information about Ireland’s Women ReBOOT programme, visit https://www.digitalskillnet.ie/news/cat-1/73/the-women-reboot-programme-free-training-for-returners-to-tech-jobs/ [accessed 27 February 2026].

13 For more information about Finland’s adult learning programmes, visit https://www.norden.org/en/info-norden/adult-education-finland [accessed 27 February 2026].

14 For more information about Singapore’s AI-powered One Talent Gateway, see Tan (2023).

15 The homepage of Canada’s Future Skills Centre (FSC) is at https://fsc-ccf.ca/ [accessed 27 February 2026].
