# Supplementary material for: Understanding Europe’s skills shortage: Patterns of participation in formal and non-formal education and training
Source: Int Rev Educ. 2026 Mar 22;72(2):231–57. doi: 10.1007/s11159-025-10175-0 (PMC13124880; doi:10.1007/s11159-025-10175-0)
Supplement: Supplementary file 1 — Supplementary file1 (PDF 432 KB) [file 11159_2025_10175_MOESM1_ESM.pdf]

Sarah Pemberton-Frings & Sonia Ilie

Understanding Europe's skills shortage: Patterns of participation in formal and non-formal education and training

Supplementary Online Appendix

https://doi.org/10.1007/s11159-025-10175-0 *Int Rev Educ* (2026)

SOA Table 1 Variables and coding decisions

Variable	Coding decisions
Outcomes of interest	
Education or training received during previous 12 months	educ12months: binary variable where respondent has participated in either formal or non-formal education (1) or not (0) in the last 12 months<br>Notes: used in 2022 regression
Education or training received during previous 4 weeks	educ4weeks: binary variable where respondent has participated in either formal or non-formal education (1) or not (0) in the last 4 weeks<br>Notes: used in descriptive statistics to measure participation in 2013–2022; used in regression for 2013–2022
Job-related, non-formal education or training during previous 12 months	Uses educnfe12=1, where educnfe12 is the variable measuring participation in non-formal education and training in the last 12 months, with answer options: (1) Participating in at least one job-related non-formal education or training activity; (2) Participating only in non-job-related/personal nonformal education or training activities; (3) Not participating in any non-formal education or training activity<br>Notes: used in 2022 regression
Job-related, non-formal education or training during previous 4 weeks	Uses educnfe4=1, where educnfe4 is the variable measuring participation in non-formal education and training with answer options: (1) Participating in at least one job-related non-formal education or training activity; (2) Participating only in non-job-related/personal nonformal education or training activities; (3) Not participating in any non-formal education or training activity<br>Notes: used in 2021 comparison to 2022, as educnfe12 not available for 2021
Non-formal education or training during previous 4 weeks	Uses educnfe4=4, where educnfe4 is derived from couratt (Did you attend any courses, seminars, conferences or received private lessons or instructions outside the regular education system within the last 4 weeks). With educnfe4(4)=couratt(1): Yes; and educnfe4(3)=couratt(2): No.<br>Notes: used in 2013–2022 comparison, as educnfe4 new coding began in 2021. In this comparison, for 2021–2022 educnfe4=1 and educnfe4=2 were combined, to be comparable to 2013–2020 educnfe4=4.
Personal characteristics	
Sex	sex: (1) Male, (2) Female

Age	Some countries have age available in years, others have age only in 5-year age bands. To ensure consistency across, the variable AGE_5groups was created with age grouped in 16–24, 25–34, 35–44, 45–54, and 55–64.<br>As some countries did not have an ‘age’ continuous variable (only banded), we did not add an age-squared term even though we hypotheses that age may not have a linear relationship with participation in education or training.
Education	Uses hatlev1d, which is the level of education according to ISCED 2011, with 3 levels: lower secondary, upper secondary, and third level.
Child	Uses hhchildr, which measures the ‘existence of children in the same household’: (1) child(ren) of the person live(s) in the same household; (2) child(ren) of the person do(es) not live in the same household
Partner	Uses hhpartnr, which measures the ‘existence of spouse/co-habiting partner in the household’: (1) The spouse or cohabiting partner of the person lives in the same household; (2) The spouse or cohabiting partner (if any) of the person does not live in the same household
Urbanisation	Uses degurba, which measures ‘degree of urbanisation’: (1) Cities; (2) Towns and suburbs; (3) Rural areas
Job-related characteristics	
Tenure	Uses startime, which is ‘time in months since the person started current employment’. Aggregated to: (1) <1 year; (2) Between 1 and 5 years; (3) Between 5 and 10 years; (4) 10 years or more
Job type	Uses ftpt, which is ‘full- or part-time main job (self-defined)’: (1) full-time job; (2) part-time job
Temporary/permanent	Uses temp, which is ‘permanency of main job’: (1) permanent job; (2) fixed-term job
Occupation	Uses isco08_1d, the 1-digit occupation code of ISCO-08: (0) Armed Forces Occupations; (10) Managers; (20) Professionals; (30) Technicians and Associate Professionals; (40) Clerical Support Workers; (50) Service and Sales Workers; (60) Skilled Agricultural, Forestry and Fishery Workers; (70) Craft and Related Trades Workers; (80) Plant and Machine Operators, and Assemblers; (90) Elementary Occupations<br>Reference value: (20) Professionals
Occupation skill level	Aggregates occupation into four groups: (0) Armed forces occupations; (1) Low skill (Elementary Occupations); (2) mid-skill (Clerical Support Workers; Service and Sales Workers; Skilled Agricultural, Forestry and Fishery Workers; Craft and Related Trades Workers; Plant and Machine Operators, and Assemblers); (3) high-skill (Managers; Professionals; Technicians and Associate Professionals)<br>Reference value: (2) mid-skill
Industry	Uses nace2_1d, the 1-digit economic activity code from NACE Rev. 2: (1) Agriculture, forestry and fishing; (2) Mining and quarrying; (3) Manufacturing; (4) Electricity, gas, steam and air conditioning supply; (5) Water supply; sewerage; waste management and remediation activities; (6) Construction; (7) Wholesale and retail trade; repair of motor vehicles and motorcycles; (8) Transporting and storage; (9) Accommodation and food service activities; (10) Information and communication; (11) Financial and insurance activities; (12) Real estate activities; (13) Professional, scientific and technical activities; (14) Administrative and support service activities; (15) Public

	administration and defence; compulsory social security; (16) Education; (17) Human health and social work activities; (18) Arts, entertainment and recreation; (19) Other services activities; (20) Activities of households as employers; undifferentiated goods- and services-producing activities of households for own use; (21) Activities of extraterritorial organisations and bodies.<br>Reference value: (16) Education
Industry with high skills mismatch	Binary variable using industry: (1) high skills mismatch industries (Professional services, Information and communication, Finance & insurance, Mining & quarrying, Education, Energy supply services, and Public sector & defence); (0) all other industries.
Work from home	Uses homework, which measures 'working at home for the main job': (1) Person mainly works at home; (2) Person sometimes works at home; (3) Person never works at home
Supervisory responsibilities in main job	Uses supvisor, which measures 'Supervisory responsibilities in main job': (1) yes; (2) no
Number of jobs	Uses numjob, which measures 'number of jobs': (1) Only one job; (2) Two jobs; (3) Three jobs or more
Size of the local unit for main job	Uses sizefirm, 'size of the local unit for main job', aggregated to: (1) <10 people; (2) 10–19 people; (3) 20–49 people; (4) 50–249 people; (5) 250 people or more
Hours worked per week	Uses hwusual and hwusu2j, which measures number of hours per week usually worked in main job and Number of hours per week usually worked in second job. Summed the hours worked shown by the two variables and aggregated into 4 groups: (1) <30 hours; (2) 30–50 hours; (3) 50–80 hours; (4) 80+ hours

SOA Table 2 Commentary on gender differences in participation rates for different types of learning in select European countries in 2022

**Type of learning**	**Commentary**
All education and training	In five countries, female participation rates outpaced that of males by more than 10 percentage points: Latvia (15% men, 26% women), Finland (39% men, 49% women), Estonia (47% men, 57% women), Iceland (47% men, 60% women), and Sweden (53% men, 65% women). Three countries stood at the other end of the spectrum: Slovakia (47% men, 41% women), the Czech Republic (25% men, 23% women), and Italy (22% men, 20% women).
Formal education and training	Only three countries displayed participation rates over 10% for men, whereas six countries had female participation rates over 10%, peaking at 22% for Iceland. On average across European countries for 2022, female participation rates were 1.6 percentage points higher than those of males.
Non-formal education and training	The gender difference was higher than for formal education and training, with female participation rates on average 2.6 percentage points higher than males. Like all learning, countries with the largest disparities are Latvia (15% men,

	25% women), Iceland (40% men, 50% women), and Estonia (45% men, 57% women). Also, like the trends seen in all learning, Slovakia, the Netherlands, and Italy had the largest gender disparity in participation rates, with more males participating than females. Since participation rates across countries were higher in non-formal education and training than formal, non-formal trends drove those in all learning.
Non-formal, job-related education and training	Female participation rates ranged from 1% in Bulgaria to 83% in Iceland for women and 1% in Bulgaria to 84% in Iceland for men. The Iceland estimates were likely overestimated due to a survey error (scheduled to be corrected at the end of 2024). On average, female participation rates were higher than male participation rates by one percentage point, and the difference was largest for Latvia (12% of men, 21% of women), Estonia (42% of men, 50% of women), and Hungary (16% of men, 24% of women).
Non-formal, non-job-related education and training	Female participation rates ranged from 0% in Bulgaria to 24% in Denmark, and male participation rates ranged from 0% in multiple countries to 16% in Iceland. On average, female participation rates outpaced males' by 1.3 percentage points.

SOA Table 3 Formal and non-formal job-related training rates by occupation in selected European countries in 2022, weighted % (formal/non-formal)

	Highly skilled			Medium-skilled					Low-skilled	Other
**Countries**	Managers	Professionals	Technicians and Associate Professionals	Clerical Support Workers	Service and Sales Workers	Skilled Agricultural, Forestry and Fishery Workers	Craft and Related Trades Workers	Plant and Machine Operators, and Assemblers	Elementary Occupations	Armed Forces Occupations
AT	4.5/38.7	12.2/49.0	6.8/37.3	3.2/26.8	3.7/21.9	0.4/30.3	2.3/18.4	0.2/20.9	1.4/6.6	0.0/66.7
BE	1.4/29.3	5.1/32.0	2.6/26.8	1.9/16.9	2.2/15.9	0.0/17.5	0.8/11.9	1.8/13.3	1.3/7.8	1.1/21.1
BG	0.8/2.7	1.9/3.8	2.2/1.7	2.1/1.6	1.2/1.2	0.9/0.3	0.6/0.3	0.4/0.7	0.1/0.1	1.3/2.5
CH	8.5/45.2	13.4/58.6	10.7/41.3	6.5/25.1	6.9/28.8	5.3/29.8	5.2/21.5	3.4/28.9	1.9/11.2	sup./sup.
CY	3.2/42.0	6.1/46.5	5.4/29.2	3.6/21.4	4.1/16.6	4.2/8.3	1.2/10.1	1.8/12.0	0.7/5.1	6.6/17.1
CZ	1.3/29.8	3.4/37.2	1.4/36.1	1.3/25.7	1.2/21.0	0.0/13.8	0.1/17.9	0.3/23.3	0.2/12.4	10.8/50.0
DE	1.8/17.1	7.1/26.5	4.3/17.5	3.3/9.4	4.0/8.4	2.0/9.3	2.3/7.0	0.9/5.5	1.5/2.3	6.4/17.1
DK	4.8/27.4	8.7/34.6	8.6/29.4	13.3/21.8	12.2/28.2	8.1/29.5	7.0/20.2	1.9/24.8	6.5/16.4	25.0/47.1
EE	7.8/67.8	10.7/71.5	8.3/57.9	10.0/43.0	8.4/48.1	5.6/35.2	4.3/32.8	1.7/33.3	3.4/27.1	25.0/75.0
EL	1.8/4.5	4.3/7.7	2.7/5.6	3.0/1.8	1.7/1.9	0.3/0.5	1.0/1.5	0.7/0.6	1.3/0.4	0.8/2.2
ES	4.5/27.2	8.0/43.7	5.4/31.4	6.1/29.8	4.3/20.9	0.7/11.5	1.1/17.5	1.5/15.8	1.7/12.0	10.6/41.9
FI	10.9/41.7	16.6/38.9	16.4/37.0	13.6/20.1	13.1/29.1	10.5/26.1	5.2/21.8	6.5/22.7	12.0/15.5	sup./sup.
FR	1.9/35.7	5.1/45.7	2.9/36.8	3.4/29.7	3.3/27.2	0.4/22.6	1.0/23.3	0.9/24.5	1.4/17.2	2.7/44.6
HR	1.8/12.1	2.7/18.8	3.3/10.2	2.6/4.7	0.7/2.7	0.7/2.6	0.5/2.0	0.2/1.5	1.2/0.5	0.0/9.7
HU	1.2/23.5	3.0/37.8	1.6/33.6	1.2/22.4	0.8/20.9	0.5/9.2	0.3/12.0	0.1/13.5	0.2/17.4	0.0/77.8
IE	5.6/14.6	8.8/20.2	7.3/15.6	7.3/10.9	8.6/12.0	0.7/10.3	3.1/10.0	4.2/11.4	5.4/5.7	sup./sup.
IS	10.3/90.7	19.1/92.6	16.2/86.8	17.2/77.8	24.0/80.6	8.8/94.4	12.2/87.0	4.8/78.3	11.0/71.4	sup./sup
IT	0.7/21.5	4.1/42.2	2.9/33.3	1.9/23.5	1.8/15.0	0.4/15.3	0.3/15.2	0.4/15.7	0.6/9.2	2.0/26.1
LT	1.8/29.1	2.7/34.2	3.9/26.3	1.9/23.6	1.8/13.9	1.7/5.9	0.7/6.3	0.2/6.7	1.3/3.4	6.5/41.9
LU	4.6/35.4	7.3/44.9	5.7/34.3	5.6/19.1	7.9/22.4	0.0/23.1	4.0/19.5	1.0/14.3	1.4/7.3	sup./sup.

	Highly skilled			Medium-skilled					Low-skilled	Other
**Countries**	Managers	Professionals	Technicians and Associate Professionals	Clerical Support Workers	Service and Sales Workers	Skilled Agricultural, Forestry and Fishery Workers	Craft and Related Trades Workers	Plant and Machine Operators, and Assemblers	Elementary Occupations	Armed Forces Occupations
LV	3.3/33.4	7.8/39.6	4.7/28.8	0.8/17.5	2.3/10.8	0.0/6.7	1.2/3.8	0.0/7.0	0.2/3.3	6.3/28.6
MT	7.1/58.8	17.4/59.4	8.2/45.9	7.7/37.4	7.4/35.8	0.0/11.1	2.2/16.1	0.7/11.1	2.3/25.0	10.0/75.0
NL	9.8/29.4	13.7/37.3	11.2/33.1	8.0/20.6	11.8/25.0	6.0/24.5	5.2/23.7	6.0/31.0	6.0/12.7	15.7/42.9
NO	5.4/53.8	12.2/57.1	7.3/50.6	7.9/39.2	13.8/38.8	5.1/35.3	6.3/39.3	3.7/31.6	11.9/29.7	28.0/68.0
Pl	1.0/22.4	2.8/27.4	1.9/20.8	2.0/14.7	1.2/10.5	0.2/4.2	0.3/7.3	0.4/8.0	0.5/5.0	3.3/38.6
PT	3.8/38.1	6.6/53.8	3.4/40.5	3.8/28.8	2.1/23.5	1.7/15.9	2.0/18.2	1.0/19.7	1.6/10.3	1.6/42.1
RO	0.4/13.3	0.8/18.5	0.7/15.0	0.5/11.6	0.4/10.4	0.1/1.3	0.2/10.2	0.1/11.4	0.3/8.0	0.6/14.8
SE	6.1/54.2	12.5/52.1	7.9/47.5	9.4/35.9	15.7/36.2	6.2/31.0	3.7/30.9	4.3/34.0	11.7/17.5	14.3/53.4
SI	4.8/47.7	7.3/54.1	5.4/37.4	6.2/30.1	5.3/26.3	1.6/22.9	2.0/16.9	1.9/20.2	2.1/12.4	8.9/43.8
SK	1.4/57.6	1.9/58.2	1.2/61.7	0.6/59.6	1.1/44.7	0.0/34.7	0.3/42.8	0.1/54.7	0.0/41.3	0.0/58.2

Notes: Yellow shading indicates highest rates of training, blue shading indicates lowest, and 'sup' denotes suppressed cells where unweighted $n < 30$.

SOA Table 4 Formal and non-formal job-related training rates by industry (abbreviated) in selected European countries in 2022, weighted % (formal/non-formal)

	Highest reported upskilling need																				Lowest reported upskilling need
**Countries**	Professional, scientific and technical activities	Information and communication	Financial and insurance activities	Mining and quarrying	Education	Electricity, gas, steam and air conditioning	Public administration and defence	Real estate activities	Human health and social work activities	Construction	Arts, entertainment and recreation	Manufacturing	Other services activities	Wholesale and retail trade	Agriculture, forestry and fishing	Administrative and support activities	Water supply & waste management	Transporting and storage	Accommodation and food service activities	Activities of households as employers	Extraterritorial organisations
Great[a]	20	20	17	27	16	17	16	14	18	15	17	15	16	15	15	14	14	14	14		
Mod[a]	53	52	52	42	51	50	50	51	47	49	46	47	46	45	44	44	43	42	41		
AT	9.1/36.4	7.0/37.5	5.4/49.8	sup./sup.	17.8/48.3	5.6/33.3	4.9/40.1	10.7/24.0	6.6/46.9	2.2/18.3	9.2/26.0	3.6/25.6	4.6/27.2	3.6/22.8	0.0/27.7	3.5/18.9	0.0/23.3	3.2/26.9	4.3/12.3	sup./sup.	sup./sup.
BE	4.3/29.6	3.9/27.0	0.9/33.0	sup./sup.	5.1/28.7	4.6/30.5	2.7/24.9	3.9/26.1	5.1/26.0	0.3/15.4	2.7/16.4	1.9/18.6	2.2/16.6	0.8/12.6	0.0/15.8	1.7/15.4	0.0/19.1	0.7/18.0	2.2/5.9	sup./sup.	2.5/46.2
BG	3.0/1.8	2.9/4.2	4.1/1.3	0.0/0.0	1.5/4.5	1.8/0.5	1.1/3.4	0.0/2.5	1.9/3.0	0.3/0.3	1.5/3.9	0.9/0.8	2.5/2.5	0.5/0.6	0.6/0.2	1.0/1.1	0.0/2.0	1.0/1.2	0.7/0.2	sup./sup.	sup./sup.
CH	10.1/41.1	8.6/43.1	9.2/45.4	sup./sup.	18.0/64.6	8.1/54.1	8.8/53.7	6.7/24.4	12.6/52.8	6.5/26.3	11.1/41.3	6.0/28.4	8.9/43.8	6.4/28.3	3.9/29.9	6.8/23.7	8.5/30.5	5.4/35.8	5.5/16.2	5.0/11.3	5.0/37.5
CY	5.5/31.1	5.4/28.0	2.7/49.6	sup./sup.	5.9/52.3	3.1/28.1	8.3/28.0	6.5/12.9	6.6/41.5	1.6/11.5	4.8/15.9	1.1/13.2	2.4/22.0	2.6/14.6	1.2/9.8	1.5/14.9	0.0/13.0	3.5/30.1	4.4/13.4	0.0/0.0	7.7/23.1
CZ	2.0/30.3	1.6/35.2	0.4/40.3	0.0/18.0	4.9/32.9	2.6/33.8	2.4/35.7	1.9/23.0	2.4/35.1	0.3/17.5	2.2/23.9	0.5/25.7	1.3/24.3	0.8/21.5	0.0/19.7	1.2/21.9	0.0/27.3	0.3/25.7	1.0/12.9	0.0/7.6	sup./sup.
DE	6.6/22.2	7.1/16.3	2.8/21.9	0.7/6.4	9.3/27.2	3.0/17.2	4.3/19.9	3.3/16.6	5.0/23.1	2.4/7.8	6.0/10.2	2.4/9.1	3.4/12.9	3.2/8.0	1.7/10.0	2.3/6.2	2.1/13.8	2.0/8.2	4.1/3.7	1.8/2.5	sup./sup.
DK	10.0/30.6	7.8/23.9	8.3/31.6	2.8/36.1	12.7/31.4	6.6/47.3	11.4/38.1	5.2/34.4	10.9/37.1	6.2/20.9	13.8/22.3	4.9/24.1	9.9/30.9	7.0/20.5	6.7/24.6	7.2/22.1	3.5/38.0	5.6/29.0	13.3/13.9	sup./sup.	3.4/37.9
EE	11.1/64.8	6.2/56.6	9.5/72.8	3.4/51.7	12.8/75.0	12.0/72.0	14.2/78.9	3.8/31.6	10.2/70.1	5.6/38.4	7.8/61.7	5.2/37.7	9.9/55.0	4.7/45.4	6.2/32.9	7.1/37.6	7.7/51.3	2.9/41.3	6.1/30.5	sup./sup.	sup./sup.
EL	3.6/4.5	4.0/9.2	2.2/11.2	0.0/4.9	5.1/7.6	3.6/5.6	1.8/2.7	sup./sup.	4.0/5.8	1.2/2.2	5.1/3.7	1.2/1.4	1.2/1.5	2.0/2.0	0.3/0.7	1.4/1.4	0.5/0.0	0.4/3.1	1.8/1.9	0.0/0.0	sup./sup.
ES	6.9/28.0	6.1/35.1	4.1/45.9	1.2/25.0	8.0/47.2	9.2/32.4	6.4/34.8	5.0/23.1	7.2/42.6	1.1/15.9	7.2/23.2	2.7/22.1	3.5/25.1	3.7/17.6	0.6/11.8	2.9/23.3	1.6/25.7	2.3/22.0	5.5/13.9	1.9/4.2	sup./sup.
FI	13.8/33.8	14.2/26.7	11.1/30.3	sup./sup.	20.5/44.4	7.1/38.9	16.1/39.5	13.2/30.4	18.8/40.4	5.6/23.9	24.0/35.7	8.5/28.1	19.8/39.1	11.2/25.8	9.1/22.8	13.4/23.0	11.0/31.2	8.9/22.5	8.5/15.0	sup./sup.	sup./sup.
FR	4.4/39.9	2.4/33.0	4.2/46.7	sup./sup.	4.1/42.2	1.2/54.0	3.2/43.3	1.8/34.9	5.7/36.3	0.8/21.0	3.1/35.4	1.4/32.0	3.1/33.9	1.9/25.8	0.4/23.6	2.1/25.9	1.0/36.9	1.5/29.3	3.1/16.8	1.3/3.2	sup./sup.
HR	2.4/12.4	4.1/23.3	5.0/12.3	0.0/2.8	2.1/18.0	0.0/5.9	1.9/9.5	0.0/6.5	2.7/9.7	0.8/2.7	7.8/8.6	0.6/4.0	1.1/7.6	0.8/3.8	1.2/1.7	1.6/6.8	0.7/2.1	0.5/4.0	3.0/1.0	sup./sup.	sup./sup.
HU	1.7/14.1	1.6/10.7	3.1/15.6	9.1/9.1	3.1/57.5	0.0/15.4	1.5/65.0	0.0/4.1	1.0/63.3	0.0/10.4	2.0/12.1	0.6/13.3	0.0/8.3	1.5/10.9	1.3/10.7	1.4/12.7	0.0/11.2	0.4/12.6	0.7/10.3	sup./sup.	sup./sup.
IE	9.3/17.3	4.7/13.0	6.9/17.2	sup./sup.	10.6/22.7	3.6/17.4	8.6/19.3	10.1/16.5	10.7/22.2	3.8/11.9	6.8/12.5	4.6/10.2	7.0/12.4	5.1/5.1	1.3/10.5	7.6/11.1	4.3/28.3	2.4/13.9	9.3/5.0	sup./sup.	sup./sup.

	Highest reported upskilling need																				Lowest reported upskilling need
**Countries**	Professional, scientific and technical activities	Information and communication	Financial and insurance activities	Mining and quarrying	Education	Electricity, gas, steam and air conditioning	Public administration and defence	Real estate activities	Human health and social work activities	Construction	Arts, entertainment and recreation	Manufacturing	Other services activities	Wholesale and retail trade	Agriculture, forestry and fishing	Administrative and support activities	Water supply & waste management	Transporting and storage	Accommodation and food service activities	Activities of households as employers	Extraterritorial organisations
IS	12.2/91.7	8.9/88.6	9.4/89.7	sup./sup.	23.3/91.3	7.1/100.0	20.9/91.4	12.5/sup.	26.1/94.0	13.8/83.3	16.4/84.4	9.7/88.2	15.4/85.0	12.6/75.4	8.7/90.9	23.7/80.0	7.7/sup.	9.6/88.7	14.7/60.0	sup./sup.	sup./sup.
IT	2.4/37.6	2.8/35.8	1.4/44.5	0.0/21.1	3.8/36.8	3.3/34.4	2.3/31.3	1.0/17.5	5.1/37.7	0.5/17.0	6.0/23.0	0.7/18.7	1.7/17.9	1.2/15.7	0.6/11.3	1.3/16.8	0.9/22.8	1.0/21.2	1.7/10.9	0.9/1.6	1.7/37.2
LT	3.2/29.8	2.8/30.8	4.1/40.7	0.0/7.0	2.5/34.2	2.6/20.7	3.0/33.1	1.6/15.6	2.8/36.9	1.3/9.5	5.0/29.5	1.1/12.3	1.9/20.5	1.4/13.4	0.3/5.7	1.4/10.8	1.6/14.2	1.2/12.5	3.9/8.5	sup./sup.	sup./sup.
LU	4.6/15.7	5.9/37.7	5.3/36.8	sup./sup.	7.0/39.9	0.0/27.3	9.7/53.6	7.4/44.6	21.6/27.0	3.9/16.7	9.1/27.3	5.0/30.0	7.9/5.4	6.1/25.2	0.0/31.8	4.5/37.6	0.7/sup.	2.3/9.3	7.7/34.0	3.1/46.9	5.8/33.7
LV	9.4/33.1	6.2/25.9	5.1/39.9	sup./sup.	7.3/36.1	2.8/34.0	2.9/42.0	0.6/15.1	6.2/34.6	1.5/8.2	5.4/23.0	2.0/10.0	1.2/16.8	2.3/12.7	0.0/8.4	0.0/13.2	2.7/11.4	1.0/13.8	4.2/4.7	sup./sup.	sup./sup.
MT	9.6/51.9	4.3/50.4	10.0/63.1	sup./sup.	19.1/64.7	sup./sup.	10.7/50.6	6.5/32.3	11.1/39.5	4.3/12.4	8.9/51.1	5.2/26.3	6.8/33.9	3.8/32.9	0.0/25.0	6.6/33.6	7.1/35.7	4.3/35.5	4.6/35.3	sup./sup.	sup./sup.
NL	10.5/30.4	7.7/31.9	9.0/43.9	15.2/30.4	21.4/34.0	8.8/37.0	11.0/40.7	11.7/29.3	16.3/37.0	4.9/26.0	12.0/25.5	6.3/26.4	8.3/27.8	7.3/20.8	5.6/23.2	8.8/22.2	7.8/43.8	5.3/29.0	10.5/13.1	9.7/11.8	sup./sup.
NO	7.0/56.1	6.4/47.6	7.0/49.4	2.4/60.3	16.7/51.8	13.8/60.9	12.9/65.2	5.0/45.7	14.1/52.9	6.6/41.5	13.2/32.6	4.6/34.7	11.3/46.1	7.2/40.2	3.4/38.6	8.0/37.5	8.4/49.4	3.8/42.3	15.9/28.8	sup./sup.	sup./sup.
Pl	2.0/24.5	1.9/28.4	2.3/28.0	0.3/19.4	2.6/22.7	0.6/16.3	1.8/31.4	1.5/16.6	3.9/21.3	0.9/8.3	2.3/14.3	0.8/12.0	2.0/19.3	1.0/11.8	0.3/4.3	1.1/11.7	0.6/11.5	0.8/11.3	1.3/9.4	0.3/1.8	sup./sup.
PT	5.1/41.8	5.4/45.8	2.9/52.8	0.0/21.7	5.8/52.0	10.6/51.3	4.1/40.7	2.7/43.2	3.9/39.3	1.6/16.2	5.2/37.5	2.8/23.8	5.1/31.1	2.6/26.8	1.7/15.1	2.8/30.5	0.0/35.4	2.4/32.8	2.7/15.3	0.8/3.2	sup./sup.
RO	0.4/13.9	0.4/19.4	0.7/16.1	0.0/6.3	0.6/17.8	0.4/9.2	0.8/14.0	0.4/26.3	0.8/18.2	0.3/10.8	0.3/10.1	0.3/12.9	0.5/13.7	0.5/9.8	0.1/3.0	0.8/14.2	0.0/8.0	0.2/10.1	0.5/12.0	0.0/3.9	sup./sup.
SE	8.5/46.3	7.1/40.4	6.3/49.6	1.1/44.7	18.9/52.6	4.3/53.9	9.4/56.3	6.4/44.0	15.8/48.8	4.4/38.9	15.7/42.8	4.8/38.0	12.5/47.2	7.2/36.5	2.0/32.5	10.6/32.9	3.6/50.0	5.2/39.2	16.9/19.7	sup./sup.	sup./sup.
SI	8.7/44.9	7.1/45.7	6.3/62.6	0.0/31.8	6.5/53.2	2.4/46.4	5.6/48.2	8.5/34.0	7.6/45.3	2.5/16.4	5.4/37.8	3.5/25.2	7.0/37.3	4.0/28.9	2.6/20.1	4.5/21.5	1.5/26.0	3.0/25.6	3.5/17.7	sup./sup.	sup./sup.
SK	0.9/54.0	1.8/64.0	1.0/68.1	0.0/60.3	2.4/56.6	0.0/54.6	1.1/62.3	1.7/64.4	2.1/50.6	0.5/40.7	1.7/48.3	0.3/54.1	0.0/39.1	0.3/48.4	0.0/40.3	0.9/49.9	0.0/65.0	0.4/60.4	2.1/37.2	sup./sup.	sup./sup.

Notes: a % of respondents reporting a 'great' or 'moderate' need to develop their overall level of knowledge or skills to do their main job (Cedefop 2021). Yellow shading indicates highest rates of training, blue shading indicates lowest, and 'sup' denotes suppressed cells where unweighted $n < 30$.

SOA Table 5 Summary of estimated results of the logistic regression models on the probability of participating in training. Indicates in which country and in which direction (positive or negative) the coefficient of each independent variable is statistically significant.

	**All**		**Formal**		**Non-formal**		**Non-formal, job-related**		**Non-formal, non-job-related**	
	+ve effect	-ve effect	+ve effect	-ve effect	+ve effect	-ve effect	+ve effect	-ve effect	+ve effect	-ve effect
Female (Ref: Male)	AT, DE, ES, FI, FR, HU, LT, MT, SE	SK	EE, FI, MT, SE, SK	DE	AT, DE, ES, FI, FR, HU, LT, MT, SE	SK	AT, CY, DE, ES, FI, HU, LT, MT, SE	SK	DK, ES, FI, FR, NO	
Age (Ref: 25–34) 35–44		AT, CY, DE, LT		AT, DE, DK, FI, HU, IT, LT, PL, RO	EE, HU, IT	CY	EE, IT	CY, EE, FI, HU, IT, MT	LT, LV, PL, SE	
45–54	IT, SK	AT, DE, FR, LT, LU, MT		AT, BE, CY, DE, DK, EE, ES, FI, FR, HU, IT, LT, PL, RO	DE, EE, FR, IT, MT, SK	FR, MT	DE, EE, FR, IT, MT, SK	FR, MT		AT, IT, LT, PL
55–64		AT, CY, DE, FR, LT, LU, MT, NO, PL		AT, CY, DE, DK, EE, ES, FI, FR, HU, IE, IT, LT, MT, NO, PL, RO, SE	IT	AT, CY, DE, FR, LT, LU, MT	IE, IT	AT, CY, DE, FR, LU, MT		AT, CY, DE, IT, LT, PT
Educational Attainment (Ref: Lower secondary)	AT, CY, DE, IT,		DE, FI, MT	LV	AT, CY, DE, IT,		AT, CY, DE, IT,		DE, IT	IE

	**All**		**Formal**		**Non-formal**		**Non-formal, job-related**		**Non-formal, non-job-related**	
	+ve effect	-ve effect	+ve effect	-ve effect	+ve effect	-ve effect	+ve effect	-ve effect	+ve effect	-ve effect
Upper secondary	LT, LU, PT				LT, LU, PT		LT, LU, PT			
Third level	AT, CY, DE, DK, EE, ES, FR, IT, LT, LU, MT, PT		DE, FI, IT, MT	LV	AT, CY, DE, DK, EE, ES, FR, IT, LT, LU, MT, PT, RO, SE		AT, CY, DE, EE, ES, FR, IT, LT, LU, MT, PT		DE, ES, IT	IE, NO
Child at home (Ref: No child)		DE, FR, LT		AT, DE, ES, FR, IT		DE, LT		DE, IE, LT	LV	CY, ES, FR, IT
Spouse/cohabitating partner (Ref: No co-habitating partner)		DE, SK	SK	BE, DE, HU, IT, LV, PL		SK		SK		
Urbanicity (Ref: Cities) Towns/suburbs	LT	CY, DE, LV, PL, SK	EE, SK	DE, IE		CY, LT, LV, PL, SK	FI, LT	CY, LV, PL, SK	RO SK	EE PL
Rural areas	FR, HU	DE, HR, LT, LV, RO, SK	DK, SK	AT, DE, HR, LV	FR, HU	LT, LV, RO, SK	EE, FR, HU, IT	HR, LT, LV, RO, SK	AT, HR	FI, FR, PL
Tenure (Ref: <1 year) 1–5 years		CY, ES, PL		DE, FI, MT		CY, ES, PL, SK	FI	MT, PL	LV	CY, NO
5–10 years	IE	DE, ES, PL, SK		DE, HU, IT, PL, PT, SK	IE	ES, PL		MT, PL		ES, LT, NO
10+ years	FR	DE, PL		DE, EE, FI, IT, LU, MT	FR	PL	FR	MT, PL		BE, NO
Part-time (Ref: Full-time)	DE, IE	MT	DE, ES, FI, IE, NO, PT	CY, HU	DE	MT	DE	ES, MT, SE	DE, LT	CY

	**All**		**Formal**		**Non-formal**		**Non-formal, job-related**		**Non-formal, non-job-related**	
	+ve effect	-ve effect	+ve effect	-ve effect	+ve effect	-ve effect	+ve effect	-ve effect	+ve effect	-ve effect
Temporary position (Ref: Permanent)	DE, FI, PL		DE, DK, FI, FR, IT, LT		RO	BE	LT, RO	BE	HR, SK	
Occupation skill level (Ref: Mid-skill) Armed Forces Occupations	AT, DK, FI, HU, LT, PL	CY, SK	EE, PL		AT, HU, LT, PL	CY, SK	AT, FI, HU, IE, LT, LU, MT, PL	CY, IT, SK		CY
Low-skill		ES, FR, LT				FR, LT		CY, ES, FR, LT	CY, NO	
High-skill	AT, BE, CY, DE, EE, ES, FI, FR, HR, IE, IT, LT, MT, PL, PT		FI, IT, LV		AT, BE, CY, DE, EE, ES, FI, HR, IT, LT, MT, PL, PT		AT, BE, CY, DE, EE, ES, FI, HR, IT, LT, LU, MT, PL, PT		IE, LV, SK	IT
Industry with high skills mismatch (Ref: Other industries)	AT, BE, CY, DE, EE, FR, HR, HU, IT, LT, MT, PL, PT, SE		AT, CY, EE, FR		AT, CY, DE, EE, FR, HR, HU, IT, LT, MT, PL, PT		AT, CY, DE, EE, FR, HR, HU, IT, LV, PL, PT	IE	HR, IE, MT	
WFH (Ref: Mainly WFH) Some WFH	AT, CY, DK, EE, FR, IT, LV	DE	DE, LV		AT, CY, DE, EE, FI, FR,	DK	AT, CY, DE EE, FR, HU		IE, SK	AT

	**All**		**Formal**		**Non-formal**		**Non-formal, job-related**		**Non-formal, non-job-related**	
	+ve effect	-ve effect	+ve effect	-ve effect	+ve effect	-ve effect	+ve effect	-ve effect	+ve effect	-ve effect
					HU, IT, LV, SE		IT, LV, SE			
No WFH		BE, DE, DK, IE, IT, LT, PL, PT, SK		HU, PL	BE, DE, DK, IE, IT	DK, LT, PL, PT, SK	HU	BE, DE, DK, IE, IT, LT, PL, PT, SK	MT	AT, DE, FR, IT
Number of jobs (Ref: 1) 2	AT, DE, EE, FR, IT, MT, RO, SK		AT, EE, FI, LV, SE		AT, DE, EE, FR, IT, MT, RO, SK		AT, DE, EE, FR, IT, RO, SK	ES	RO	
3	AT, DE, FR, IT				AT, DE, FR, IT			DE, IT		
Employer size (Ref: <10) 10–19	DE, EE, MT		EE		DE, EE, MT		DE, EE			
20–49	DE, EE, SK		EE		DE, EE, SK		DE, EE, FR, IT, SK			RO
50–249	AT, DE, EE, FR, IT, SK		EE		AT, DE, EE, FR, IT, SK		AT, DE, EE, ES, FR, IT, SK			RO
250+	AT, DE, EE, FR, IT, MT, SK			FI	AT, DE, EE, FR, IT, MT, SK		AT, DE, EE, ES, FR, IT, SK			RO
Hours worked per week (Ref: <30 hours) 30–50 hours		EE		AT	DE, FR		DE, FR			
50–80 hours	DE				DE		DE			
80+ hours	IT	EE			IT	EE	IT			

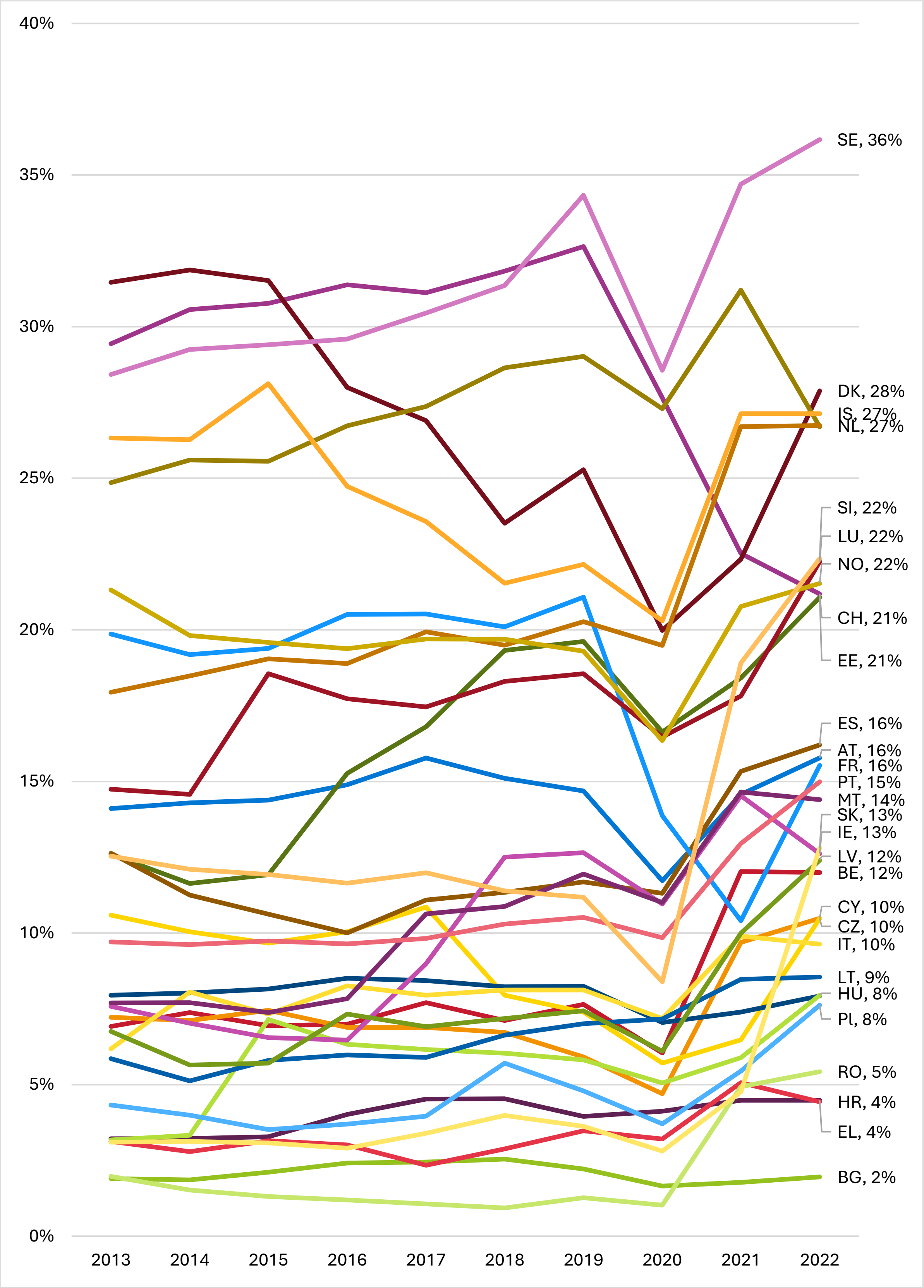

SOA Figure 1 Participation in all education and training in select European countries from 2013–2022, weighted %

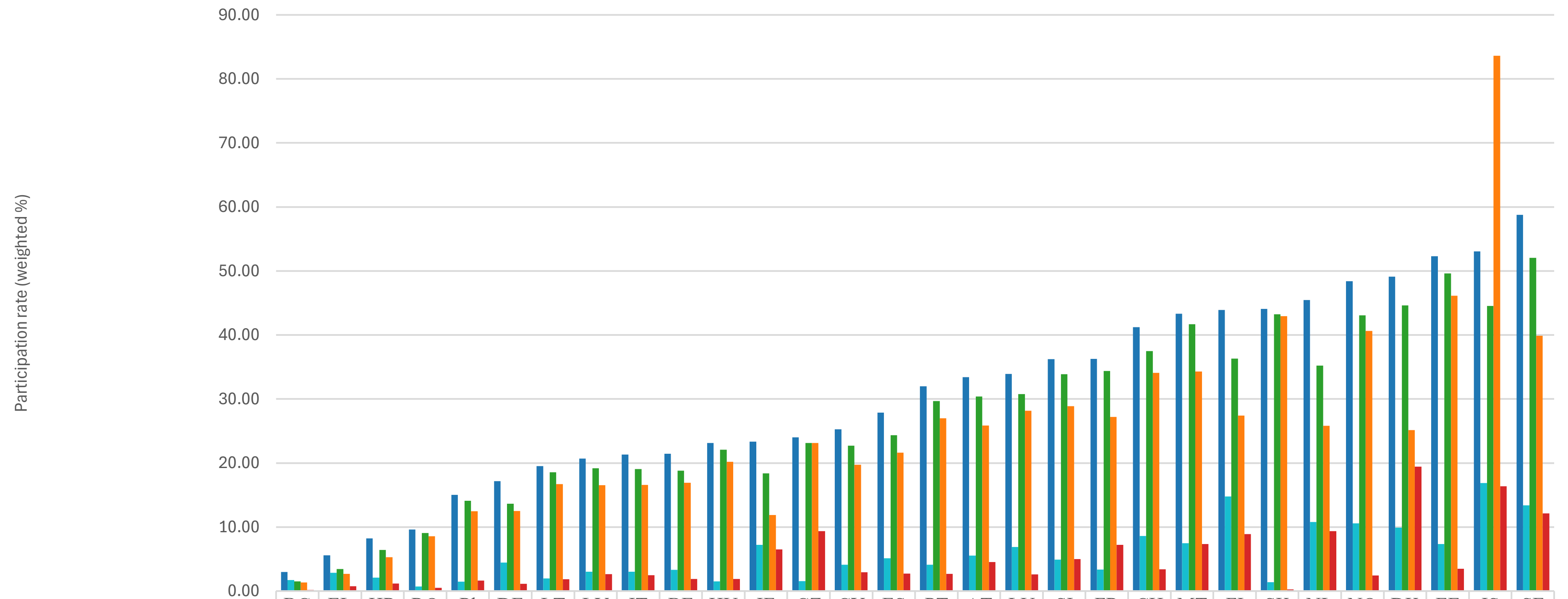

	BG	EL	HR	RO	Pl	DE	LT	LV	IT	BE	HU	IE	CZ	CY	ES
General	2.99	5.57	8.24	9.63	15.0	17.1	19.5	20.7	21.3	21.4	23.1	23.3	24.0	25.2	27.8
Formal	1.74	2.87	2.11	0.70	1.47	4.44	1.95	3.02	3.00	3.30	1.51	7.22	1.57	4.11	5.11
Non-formal	1.49	3.43	6.42	9.07	14.1	13.6	18.5	19.2	19.0	18.8	22.0	18.4	23.1	22.7	24.3
Non-formal, job-related	1.32	2.67	5.27	8.56	12.4	12.5	16.7	16.5	16.5	16.9	20.2	11.8	23.1	19.7	21.6
Non-formal, non-job-related	0.17	0.75	1.16	0.51	1.63	1.13	1.83	2.65	2.47	1.89	1.87	6.51	9.37	2.94	2.72

	PT	AT	LU	SI	FR	CH	MT	FI	SK	NL	NO	DK	EE	IS	SE
General	31.9	33.4	33.9	36.2	36.2	41.2	43.3	43.8	44.0	45.4	48.4	49.1	52.3	53.0	58.7
Formal	4.11	5.56	6.88	4.91	3.36	8.60	7.46	14.7	1.38	10.7	10.5	9.91	7.33	16.8	13.3
Non-formal	29.7	30.3	30.7	33.9	34.3	37.4	41.6	36.3	43.2	35.2	43.0	44.6	49.6	44.5	52.0
Non-formal, job-related	27.0	25.8	28.1	28.9	27.1	34.1	34.3	27.4	42.9	25.8	40.6	25.1	46.1	83.6	39.9
Non-formal, non-job-related	2.70	4.52	2.61	5.01	7.21	3.39	7.36	8.88	0.27	9.37	2.44	19.4	3.48	16.3	12.1

SOA Figure 2 Participation in different types of learning in select European countries in 2022, weighted %

Notes: Participation in formal learning activities was lower than in non-formal learning except for Bulgaria (formal: 1.7%; non-formal: 1.5%). The average participation rate for formal education was 6%, compared to 27% for non-formal learning, suggesting a possible preference or greater accessibility for non-formal learning. The discrepancy was particularly pronounced in countries such as Romania and Slovakia, where non-formal learning participation was more than ten times higher than formal learning participation. Participation rates in job-related learning far exceed those in non-job-related learning in every country. In nine of the thirty European countries in the sample, non-formal job-related participation rates were more than ten times non-job-related participation rates.

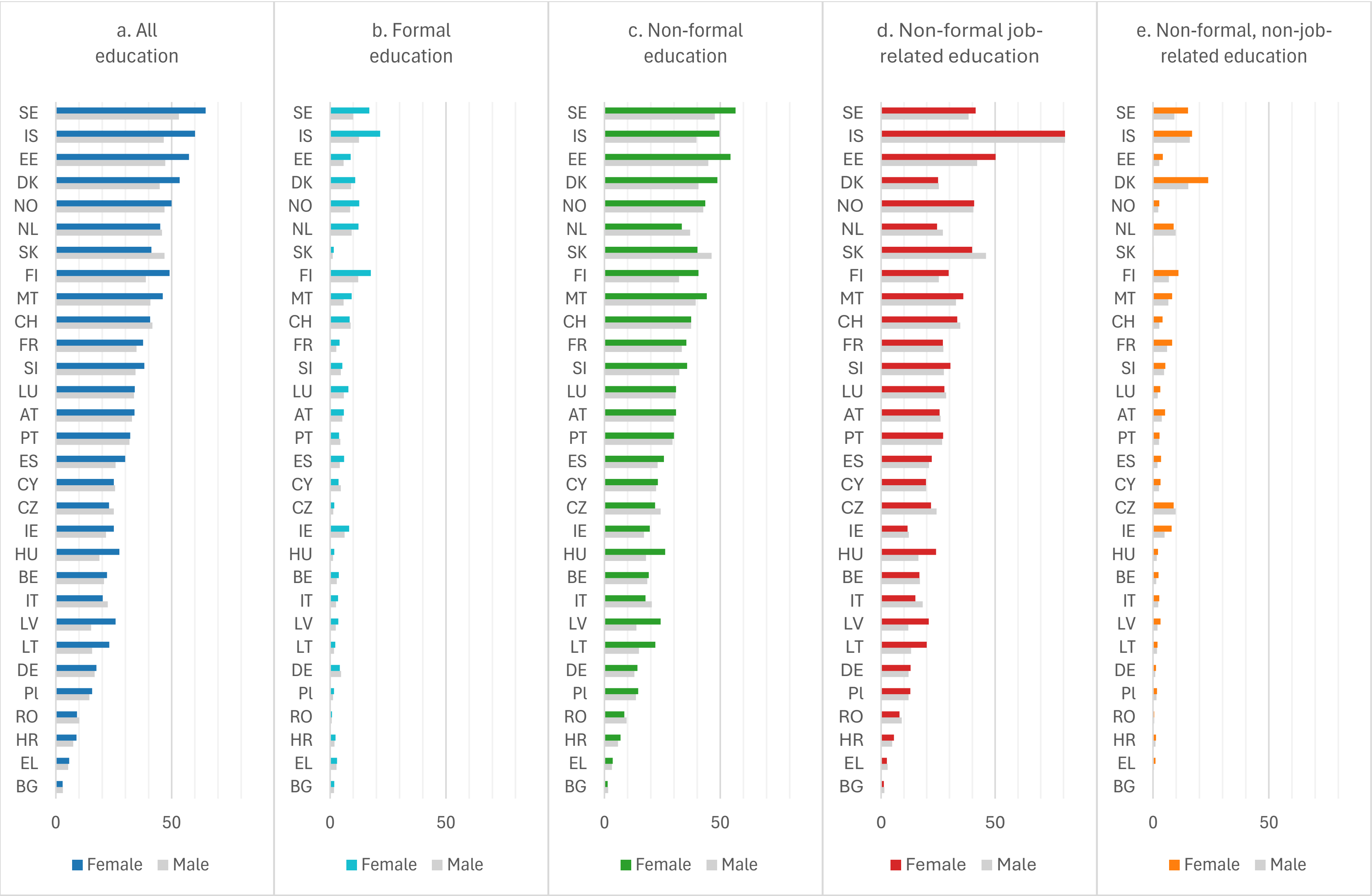

SOA Figure 3 Male and female participation in different types of education and training in selected European countries in 2022, weighted %
